# Development and Psychometric Validation of a Knowledge, Attitudes, and Practices (KAP) Questionnaire on Sustainable Diets in Taiwan

**DOI:** 10.3390/nu18060908

**Published:** 2026-03-13

**Authors:** Charlene Joy, Yu-Chih Chiang, Wen-Hwa Ko, Yi-Fang Liu

**Affiliations:** 1 Department of Nutritional Science, Fu-Jen Catholic University, New Taipei City 242062, Taiwan; 413406156@m365.fju.edu.tw; 2 Department of Restaurant, Hotel, and Institutional Management, Fu-Jen Catholic University, New Taipei City 242062, Taiwan; 141673@m365.fju.edu.tw (Y.-C.C.); 073770@mail.fju.edu.tw (W.-H.K.); 3 Ph.D. Program in Nutrition and Food Science, Fu-Jen Catholic University, New Taipei City 242062, Taiwan

**Keywords:** sustainable diet, health knowledge, attitudes, practice, psychometrics, feeding behavior, environmental health, logistic models, Taiwan

## Abstract

**Background**: Sustainable dietary transitions are increasingly emphasized in public health policy; however, validated psychometric instruments assessing knowledge, attitudes, and practices (KAP) in Asian contexts remain limited. **Objectives**: This study aimed to develop and evaluate a KAP questionnaire on sustainable diets and examine sociodemographic variations and behavioral correlates among Taiwanese adults. **Methods**: A two-phase mixed-methods design was used, including expert validation and a cross-sectional online survey (*n* = 587). Construct validity was examined using exploratory factor analysis, and reliability was evaluated through internal consistency and test–retest assessments. Multivariable logistic regression models were fitted to identify factors associated with low adherence to a low-carbon diet. Model diagnostics included variance inflation factors (VIF), Hosmer–Lemeshow tests, Nagelkerke pseudo-R^2^, and ROC analysis. **Results**: Content validity was high (S-CVI/Ave = 0.95–0.98). The attitude and practice domains demonstrated satisfactory internal consistency, whereas the knowledge domain showed comparatively lower reliability. In multivariable logistic regression analyses, higher attitude scores were independently associated with lower odds of low adherence to a low-carbon diet. Eating-out frequency and clear awareness of sustainability in Taiwan’s Dietary Guidelines were also significantly associated with adherence. The fully adjusted model demonstrated adequate calibration and excellent discrimination (AUC = 0.778). **Conclusions**: The instrument provides preliminary psychometric evidence supporting the assessment of sustainable diet-related KAP among Taiwanese adults. Attitudes appear more strongly associated with dietary practices than knowledge alone. The questionnaire may support future monitoring and research.

## 1. Introduction

Global food systems contribute substantially to greenhouse gas emissions, resource depletion, and ecological degradation [1,2]. Dietary transitions are therefore increasingly recognized as a critical pathway to advancing both environmental sustainability and population health [3]. Sustainable diets, as defined by the Food and Agriculture Organization (FAO), aim to promote health while minimizing environmental impact and respecting cultural and economic dimensions of food systems [4]. Beyond their environmental benefits, sustainable diets are closely linked to nutritional quality, often emphasizing plant-based foods that are associated with reduced risks of non-communicable diseases [5]. Assessing population readiness to adopt sustainable dietary patterns is therefore a priority for both environmental sustainability and public health nutrition.

Although interest in sustainable diets has grown rapidly in high-income countries, empirical evidence from Asian settings remains limited [6,7]. Taiwan presents a distinctive dietary context characterized by a high frequency of eating out and strong reliance on commercial food services, as documented in national surveys [8]. Sustainability concepts were incorporated into the revised Taiwan Dietary Guidelines in 2018; however, population-level data on sustainable dietary knowledge and behaviors remain scarce [9]. In addition, most existing instruments assessing sustainable diet-related knowledge, attitudes, and practices have been developed in Western populations and may not fully capture region-specific dietary patterns, food environments, and sociocultural determinants of eating behavior in East Asian contexts [10,11]. These contextual features underscore the need for culturally adapted and psychometrically robust measurement tools.

Knowledge, Attitudes, and Practices (KAP) frameworks are widely used in public health research to examine behavioral determinants and to evaluate readiness for health-related interventions [12]. However, a culturally adapted KAP instrument specific to sustainable diets in Taiwan is currently lacking [13]. Accordingly, this study aimed to: (1) develop and validate a sustainable diet KAP questionnaire among Taiwanese adults; (2) examine sociodemographic variations in KAP scores; and (3) examine associations between KAP scores, eating-out patterns, awareness of sustainability in Taiwan’s Dietary Guidelines, and low-carbon dietary adherence.

## 2. Materials and Methods

### 2.1. Study Design

A two-phase instrument development and validation study was conducted. Phase I focused on questionnaire development and psychometric evaluation. Phase II employed a cross-sectional online survey to examine behavioral correlates and sociodemographic patterns of sustainable diet practices.

### 2.2. Development of the KAP Questionnaire (Phase I)

Questionnaire items were developed based on the (1) FAO principles of sustainable diets; (2) EAT-Lancet dietary recommendations; (3) systematic reviews on environmental nutrition literacy, and (4) Taiwan’s Dietary Guidelines. The preliminary item pool comprised three conceptual domains: knowledge (factual and conceptual understanding), attitude (beliefs, values, and perceived importance), and practice (self-reported frequency of sustainable dietary behaviors). Items were formulated to reflect culturally relevant dietary patterns and food environments in Taiwan.

### 2.3. Expert Validation and Content Validity Index (CVI)

Two independent experts in nutrition and public health evaluated each item for relevance, clarity, and content representativeness. Content validity was assessed using the item-level content validity index (I-CVI) and scale-level content validity indices (S-CVI/Ave and S-CVI/UA). Following established methodological recommendations [14,15], acceptable thresholds were predefined as: I-CVI ≥ 0.78 and SCVI/Ave ≥ 0.90. The S-CVI/Ave ranged from 0.95 to 0.98, and the S-CVI/UA ranged from 0.88 to 1.00, indicating high content validity. All items met retention criteria.

### 2.4. Pilot Testing and Psychometric Evaluation

#### 2.4.1. Exploratory Factor Analysis

Exploratory factor analysis (EFA) was conducted separately for the knowledge, attitude, and practice domains using principal axis factoring to examine their underlying factor structures. Sampling adequacy was assessed using the Kaiser–Meyer–Olkin (KMO) measure and Bartlett’s test of sphericity prior to factor extraction. Orthogonal varimax rotation was applied to enhance the interpretability of factor loadings. Factor retention was guided by eigenvalues greater than 1.0 and inspection of the scree plot, in conjunction with theoretical interpretability.

#### 2.4.2. Internal Consistency

Internal consistency was assessed using Cronbach’s alpha for the Attitude and Practice domains, and Guttman’s lambda for the Knowledge domain. The Attitude domain showed strong internal consistency (α = 0.853), while the Practice domain demonstrated acceptable reliability (α = 0.834). The knowledge domain exhibited acceptable item loadings, although heterogeneity was observed, consistent with its multidimensional conceptual structure.

#### 2.4.3. Test–Retest Reliability

Temporal stability was assessed in a subsample (*n* = 63) who completed the questionnaire twice within a 14-day interval. For the knowledge domain, which consisted of categorical items, agreement between test and retest responses was evaluated using Cohen’s kappa (κ). For the attitude and practice domains, which were treated as continuous variables, test–retest reliability was assessed using the intraclass correlation coefficient (ICC) based on a two-way mixed-effects model with absolute agreement (ICC [1,3]) [16].

Items demonstrating unsatisfactory temporal stability, defined as Cohen’s kappa < 0.30, non-estimable kappa due to lack of response variability, or ICC 95% confidence intervals crossing zero, were flagged for further psychometric screening in subsequent scoring procedures. Interpretation of reliability coefficients followed commonly accepted thresholds for kappa and ICC statistics [17,18].

A subsample of 63 participants is consistent with methodological recommendations for test–retest reliability assessment in psychometric validation studies, where sample sizes ranging from 30 to 50 are generally considered adequate for estimating stability coefficients while balancing feasibility and measurement precision.

### 2.5. Scoring and Psychometric Screening

To ensure psychometric rigor and score stability, item inclusion in composite domain scores was determined based on temporal stability and response distribution characteristics. Items flagged in the test–retest analysis (κ < 0.30, ICC 95% CI crossing zero, or non-estimable κ) and/or exhibiting substantial ceiling effects (>80% agreement with limited variance) were excluded from composite domain scores due to unsatisfactory reliability and/or ceiling effects.

Excluded items were retained for descriptive reporting to preserve conceptual and policy relevance. Two knowledge items and two attitude items met exclusion criteria and were therefore omitted from inferential modeling but included in descriptive analyses. This decision was made to enhance score variability and minimize attenuation bias in subsequent regression modeling.

### 2.6. Nationwide Survey Implementation (Phase II)

#### 2.6.1. Participants and Sampling

Adults aged ≥18 years residing in Taiwan were recruited between December 2024 and February 2025 using a non-probability convenience sampling approach. The online survey was disseminated through social media platforms, university mailing lists, and community networks to maximize geographic coverage across Taiwan. Participation was voluntary and anonymous. Inclusion criteria required participants to be aged 18 years or older and currently residing in Taiwan. Responses with substantial missing data (>20% incomplete items) or implausible response patterns were excluded during data screening. After quality control procedures, a total of 587 valid responses were included in the final analysis. Given the non-probability sampling design, no post-stratification weighting was applied. Findings should therefore be interpreted as reflective of the sample surveyed rather than nationally representative estimates.

#### 2.6.2. Measures

Sociodemographic variables included age, gender, education level, occupation, residential area, and monthly income. Eating-out behaviors were assessed by self-reported dining frequency (≤1 time/day, 2 times/day, ≥3 times/day) and meal categories (breakfast, lunch, dinner, snacks/supper). Policy awareness was assessed by asking participants whether they had heard of or read Taiwan’s Dietary Guidelines and whether they were aware that the guidelines incorporate sustainability considerations. Low adherence to a low-carbon diet was operationalized based on the practice domain score (see Section 2.5).

### 2.7. Statistical Analysis

Descriptive statistics were presented as mean ± standard deviation (SD) for continuous variables and frequency (percentage) for categorical variables. Group differences in KAP scores were examined using independent-samples t-tests (for two-group comparisons) and one-way analysis of variance (ANOVA) for comparisons across three or more groups. Post hoc comparisons were performed using Bonferroni correction where appropriate. Assumptions of normality and homogeneity of variance were assessed prior to analysis.

#### 2.7.1. Logistic Regression Modeling

Multivariable logistic regression analyses were conducted to examine factors associated with low adherence to a low-carbon diet, defined as a practice score ≤ 2. This threshold corresponds to an average response between “never” and “rarely,” representing consistently low engagement rather than a percentile-based classification. Sequential models were constructed to assess incremental explanatory contributions of variable blocks:

Model 1 included education level, monthly income, sustainability awareness, and eating-out frequency.

Model 2 additionally adjusted for age and gender.

Model 3 further included residential area, occupation, knowledge score, and attitude score.

All predictors were entered simultaneously within each model block. Adjusted odds ratios (ORs) and 95% confidence intervals (CIs) were reported.

#### 2.7.2. Model Diagnostics

Multicollinearity was assessed using variance inflation factors (VIF), with values < 5 considered indicative of no problematic collinearity. Model calibration was evaluated using the Hosmer–Lemeshow goodness-of-fit test. Explanatory power was assessed using Nagelkerke pseudo-R^2^. Discriminatory capacity was evaluated using receiver operating characteristic (ROC) curve analysis and the area under the curve (AUC). The linearity of the logit assumption for continuous predictors was examined using the Box–Tidwell procedure. Where evidence of non-linearity was observed, variables were categorized accordingly. Age was entered as a categorical variable in the final model to address potential non-linearity. All statistical analyses were performed using SPSS version 29.0 (IBM Corp., Armonk, NY, USA). Statistical significance was defined as *p* < 0.05 (two-sided).

## 3. Results

### 3.1. Construct Validity and Reliability of the KAP Instrument

Exploratory factor analysis was conducted separately for the knowledge, attitude, and practice (KAP) domains using principal axis factoring with varimax rotation. The Kaiser–Meyer–Olkin (KMO) measure of sampling adequacy was 0.874, indicating meritorious suitability for factor analysis. Bartlett’s test of sphericity was significant (χ^2^ = 3756.097, *p* < 0.001), confirming that the correlation matrix was appropriate for factor extraction. Based on eigenvalues greater than 1 and inspection of the scree plot, three factors were retained, corresponding to the predefined knowledge, attitude, and practice domains. Together, these factors explained 42.96% of the total variance.

Items are loaded primarily on their respective conceptual domains. Standardized factor loadings ranged from 0.139 to 0.671 for knowledge items, 0.556 to 0.786 for attitude items, and 0.628 to 0.805 for practice items (Table 1). The knowledge domain exhibited greater heterogeneity in loadings, consistent with its multidimensional content structure. Internal consistency analysis indicated acceptable reliability for the attitude (Cronbach’s α = 0.853) and practice (Cronbach’s α = 0.834) domains. The knowledge domain demonstrated lower internal consistency (Guttman’s λ = 0.576), reflecting its broader content coverage rather than strict unidimensionality.

Convergent validity indices demonstrated stronger performance for the attitude and practice domains, whereas the knowledge domain exhibited comparatively lower average variance extracted (AVE = 0.266). This pattern is interpreted in light of the knowledge domain’s conceptual role as a content-based construct rather than a reflective latent factor. Detailed item-level response distributions for the knowledge, attitude, and practice domains are presented in Supplementary Tables S1–S3. Additional psychometric and model diagnostics are provided in Supplementary Tables S4–S6.

### 3.2. Test–Retest Reliability

Item-level test–retest reliability results for the knowledge, attitude, and practice domains are presented in Table 2. For the knowledge domain, Cohen’s kappa coefficients among estimable items ranged from 0.174 to 0.659. While several items demonstrated moderate to substantial agreement (κ ≥ 0.41), some items exhibited lower agreement, primarily attributable to limited response variability and ceiling effects rather than response inconsistency. Item K6 showed no response variability across administrations, precluding estimation of Cohen’s kappa.

For the attitude domain, intraclass correlation coefficients ranged from 0.337 to 0.623. Two attitude items (A2 and A7) demonstrated unsatisfactory reliability, as indicated by 95% confidence intervals crossing zero, and were therefore excluded from composite score calculation. The practice domain demonstrated comparatively stronger temporal stability, with ICC values ranging from 0.497 to 0.793, indicating moderate to good reliability across items. Overall, these findings support acceptable temporal stability of the instrument for population-level assessment, while highlighting the conceptual breadth and heterogeneity inherent in the knowledge domain.

In addition to item-level stability, composite domain scores demonstrated moderate temporal stability for the attitude and practice domains, consistent with the observed item-level ICC patterns. The knowledge composite score showed comparatively lower stability, reflecting the multidimensional nature of the construct.

### 3.3. Participant Characteristics

A total of 587 participants were included in the analysis. The distribution of sociodemographic and lifestyle characteristics is presented in Table 3. Women comprised 61.2% of the sample (*n* = 359), while 37.1% were men (*n* = 218); 10 participants declined to report gender. Age was relatively evenly distributed across four predefined groups. Most participants had attained a college or university level of education (61.8%), and more than half were employed in full-time positions (54.2%). The majority resided in northern Taiwan (79.2%). Monthly income distribution was broadly dispersed across categories; percentages were calculated based on valid responses (excluding 52 participants who declined to report income).

Regarding lifestyle characteristics, eating out was common, with 36.3% of participants reporting eating out three or more times per day. Lunch and dinner were the most frequently reported eating-out occasions (77.3% and 68.8%, respectively). Because eating-out categories were assessed as a multiple-response item, percentages exceed 100%. With respect to policy awareness, only 22.8% of participants reported clear awareness that Taiwan’s Dietary Guidelines or Dietary Indicators include sustainability considerations, whereas 40.7% reported no awareness.

### 3.4. KAP Score Comparisons

Comparisons of knowledge, attitude, and practice (KAP) scores across sociodemographic and lifestyle characteristics are presented in Table 4. Knowledge scores differed significantly by gender and educational attainment, with women and participants with a college or higher education reporting higher knowledge scores. Knowledge scores did not differ significantly across age groups. Attitude scores demonstrated significant gradients across age, educational level, occupation, and monthly income. Older participants and those with higher socioeconomic status showed significantly higher attitude scores. Practice scores varied significantly across age groups, occupation, monthly income, and frequency of eating out. Higher practice scores were observed among older participants, non-students, individuals with higher income, and those who ate out less frequently.

No significant differences in KAP scores were observed across most residential areas or eating-out categories. These subgroup differences informed the subsequent multivariable analyses examining factors associated with low adherence to a low-carbon diet.

### 3.5. Self-Reported Sustainable Diet and Food Waste-Reduction Actions

**Figure 1:**
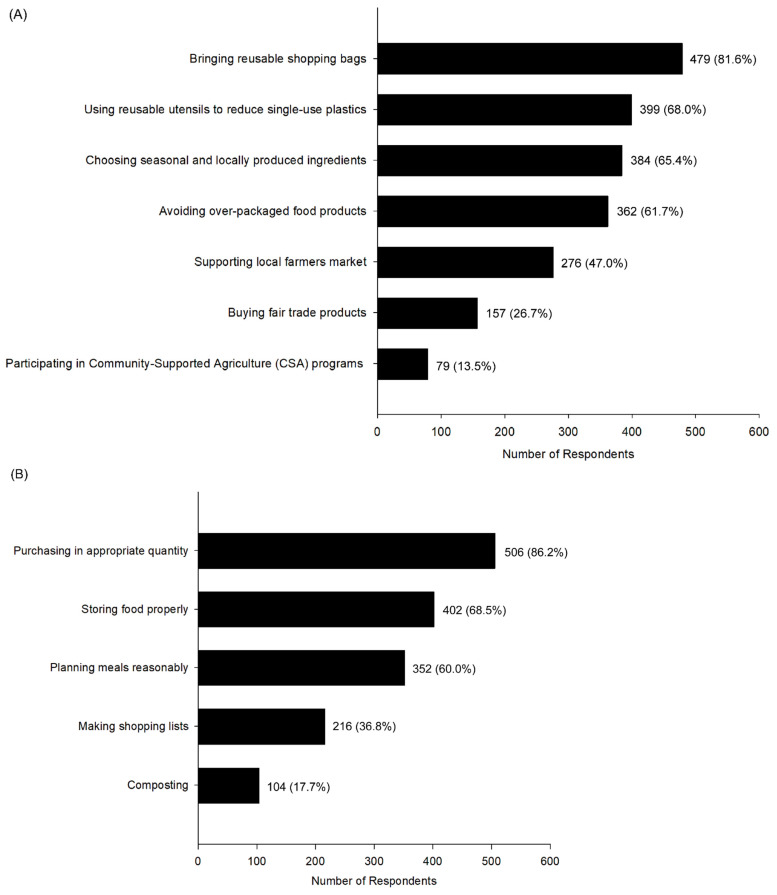
Self-reported actions undertaken by respondents to support a sustainable dietary practice (**A**) and to reduce food waste (**B**). Items were assessed as multiple-response questions; percentages represent the proportion of respondents (N = 587) selecting each action. CSA, Community-Supported Agriculture.

Figure 1 presents the distribution of self-reported actions undertaken by respondents to support a sustainable diet and to reduce food waste. These items were assessed as multiple-response questions; therefore, percentages reflect the proportion of respondents (N = 587) selecting each action. Among sustainable diet-related behaviors, bringing reusable shopping bags was the most frequently reported action (81.6%), followed by using reusable utensils to reduce single-use plastics (68.0%) and choosing seasonal and locally produced ingredients (65.4%). Avoiding over-packaged food products (61.7%) and supporting local farmers’ markets (47.0%) were also commonly reported. In contrast, participation in community-supported agriculture (CSA) programs (13.5%) and purchasing fair-trade products (26.7%) were reported by smaller proportions of respondents (Figure 1A).

Regarding food waste-reduction practices, purchasing food in appropriate quantities (86.2%) and proper food storage (68.5%) were the most frequently reported behaviors. Planning meals reasonably was reported by 60.0% of respondents, while making shopping lists (36.8%) and composting (17.7%) were less commonly endorsed (Figure 1B). These patterns may reflect greater adoption of routine, low-disruption behaviors compared with practices that require additional planning or environmental infrastructure.

### 3.6. Factors Associated with Low Adherence to a Low-Carbon Diet

Table 5 presents the results of the multivariable logistic regression analyses examining factors associated with low adherence to a low-carbon diet (practice score ≤ 2). In the fully adjusted model, all predictors were entered simultaneously. Multicollinearity diagnostics indicated no evidence of problematic collinearity (VIF range: 1.02–2.21). The Hosmer–Lemeshow goodness-of-fit test indicated adequate model calibration (*p* > 0.05). Model explanatory power increased across sequential models, with Nagelkerke pseudo-R^2^ reaching 0.246 in the fully adjusted model. ROC analysis demonstrated acceptable discrimination (AUC = 0.778), indicating a moderate ability of the model to distinguish between outcome groups. The Box–Tidwell procedure indicated no significant violations of the linearity-in-the-logit assumption for continuous predictors (all interaction terms *p* > 0.05), supporting the appropriateness of the logit specification.

In the fully adjusted model, higher attitude scores were independently associated with lower odds of low adherence to a low-carbon diet (adjusted OR = 0.915, 95% CI: 0.874–0.957, *p* < 0.001). Participants who reported eating out ≤ 2 times per day had significantly lower odds of low adherence compared with those eating out > 3 times per day (adjusted OR = 0.476, 95% CI: 0.279–0.814, *p* = 0.007). Clear awareness that Taiwan’s Dietary Guidelines incorporate sustainability considerations was also associated with lower odds of low adherence (adjusted OR = 0.373, 95% CI: 0.146–0.950, *p* = 0.039). In contrast, knowledge scores, education level, and monthly income were not independently associated with low adherence in the fully adjusted model.

## 4. Discussion

### 4.1. Overall Interpretation of KAP Structure and Measurement Performance

This study developed and evaluated a sustainable diet KAP questionnaire and examined how knowledge, attitudes, and practices were associated with adherence to a low-carbon diet among Taiwanese adults. Overall, the psychometric results support the construct validity of the attitude and practice domains, whereas the knowledge domain exhibited relatively lower internal consistency.

Exploratory factor analysis demonstrated acceptable factor loadings across domains, with strong reliability observed for the attitude and practice scales. The lower reliability of the knowledge domain (λ = 0.576) likely reflects the multidimensional and formative characteristics of sustainability literacy rather than random measurement error. Knowledge items were intentionally designed to capture conceptually distinct sustainability topics (e.g., food miles, fisheries sustainability, environmental impacts), and familiarity with one concept does not necessarily imply mastery of others. Thus, the modest internal consistency appears to reflect conceptual breadth rather than measurement weakness, consistent with prior KAP research [11,13].

Most knowledge items demonstrated acceptable test–retest reliability. Although several items exhibited ceiling effects or limited response variability, patterns commonly observed in assessments of foundational sustainability knowledge, these characteristics do not inherently indicate instability. One item showed perfect agreement across administrations, precluding estimation of Cohen’s kappa. The excellent content validity (S-CVI/Ave > 0.95) further supports the adequacy of domain coverage. Overall, the knowledge domain appears appropriate for descriptive and population-level assessment rather than fine-grained discrimination along a single latent continuum.

A recently validated Sustainable Diets Questionnaire (SDQ) similarly employed a tripartite KAP structure and formal psychometric testing in an adult population [19]. While conceptually aligned, the present instrument was specifically contextualized to Taiwanese dietary patterns, national dietary guidelines, and eating-out behaviors, which are particularly salient in East Asian urban food environments. This comparison highlights the growing international interest in validated sustainable diet KAP tools and underscores the importance of cultural adaptation when operationalizing sustainability constructs across populations.

Beyond measurement performance, the observed behavioral associations warrant careful interpretation. The inverse association between eating-out frequency and low-carbon dietary adherence is broadly consistent with prior findings linking frequent eating out to higher meat intake and lower plant-based consumption [20]. However, given the cross-sectional design, causal relationships cannot be inferred.

### 4.2. Attitudes as a Key Correlate Linking Knowledge and Practices

Across analyses, attitudes emerged as the factor most consistently associated with sustainable dietary practices. Correlation analyses demonstrated stronger attitude–practice associations across subgroups, whereas knowledge–practice correlations were comparatively weaker. Multivariable logistic regression further indicated that higher attitude scores were independently associated with lower odds of low adherence to a low-carbon diet after adjustment for sociodemographic and behavioral variables. In contrast, education level, income, and knowledge scores were not independently associated with adherence in the fully adjusted model.

Model diagnostics indicated adequate calibration (Hosmer–Lemeshow *p* > 0.05), no evidence of problematic multicollinearity (VIF < 2.5), and acceptable discriminatory performance (AUC = 0.778). The inclusion of knowledge and attitude scores in the final model substantially increased explanatory power (Nagelkerke R^2^ = 0.246). High discrimination may partly reflect conceptual proximity between attitudinal constructs and the behavioral outcome within the same KAP framework. High discrimination should therefore be interpreted within the context of shared conceptual framing between predictors and outcome.

These findings are consistent with established behavioral frameworks, including the Theory of Planned Behavior, which identifies attitudes as a proximal determinant of behavioral intention and action [21]. Prior research on sustainable consumption similarly suggests that knowledge alone may be insufficient to predict behavior in the absence of supportive motivational or normative orientations [22,23]. Collectively, the present findings indicate that attitudinal orientation may represent an important correlational factor in sustainable dietary engagement and may warrant consideration in future intervention strategies.

### 4.3. Practice Patterns and Behavioral Feasibility in Daily Life

The classification of low adherence (score ≤ 2) was based on responses corresponding to “never” or “rarely”, representing consistently low engagement rather than a distribution-based threshold. Within this framework, practice scores differed significantly across age, occupation, income, and eating-out frequency, highlighting the potential influence of structural and contextual factors. Older participants and those who reported eating out less frequently exhibited higher engagement levels, suggesting that greater food-related autonomy or routine stability may be associated with sustainable dietary practices.

Figure 1 indicates that lower-effort or habit-based behaviors (e.g., using reusable bags, purchasing appropriate quantities, proper food storage) were widely reported, whereas practices requiring greater structural or lifestyle modification (e.g., participation in community-supported agriculture programs or composting) were less prevalent. These gradients are broadly consistent with prior literature emphasizing the roles of convenience, perceived behavioral control, and environmental infrastructure in shaping sustainable food-related behaviors [24,25]. However, given the cross-sectional nature of the present study, these patterns should be interpreted as descriptive associations rather than causal determinants.

### 4.4. Policy Awareness and the Translation of Attitudes into Behavior

Clear awareness that Taiwan’s dietary guidelines incorporate sustainability considerations was independently associated with lower odds of low-carbon diet non-adherence, even after full adjustment. This association may reflect the role of policy-related knowledge in reinforcing conceptual salience and normative framing of sustainable dietary behaviors. While causal inference cannot be established, the findings are consistent with international policy frameworks that emphasize integrating sustainability principles into national dietary guidelines as a strategy to promote environmentally sustainable consumption [4]. Empirical research similarly suggests that clear policy communication may be associated with increased public engagement in sustainability-oriented behaviors [26]. Taken together, these findings underscore the potential relevance of policy visibility and messaging as correlational factors linked to sustainable dietary practice.

### 4.5. Implications for Sustainable Diet Promotion and Intervention Design

The findings suggest that efforts to promote sustainable and low-carbon diets may benefit from incorporating attitudinal components in addition to knowledge-based education. While foundational knowledge is an important prerequisite for informed decision-making, the present results indicate that attitudes were more consistently associated with behavioral adherence in this population. Intervention strategies that enhance personal relevance, perceived behavioral feasibility, and supportive social norms have been emphasized in prior behavioral research as potentially important components of sustainable diet promotion [27,28]. However, longitudinal and intervention-based studies are required to determine whether strengthening attitudes leads to measurable behavioral change.

At the institutional level, increasing the visibility and clarity of sustainability-related messaging within dietary guidance frameworks may contribute to greater public engagement. Nevertheless, the extent to which policy communication directly influences dietary practices warrants further empirical investigation. Beyond intervention design, the instrument may also support population-level monitoring of sustainable dietary engagement within national nutrition surveillance systems. Integration of culturally adapted KAP tools into routine assessment frameworks could facilitate evidence-informed policy development and evaluation.

### 4.6. Strengths and Considerations

This study integrates instrument development, psychometric evaluation, subgroup comparisons, and multivariable modeling within a unified KAP framework. The inclusion of formal model diagnostics (e.g., multicollinearity assessment, calibration testing, discrimination analysis, and linearity evaluation) enhances analytical transparency and supports the robustness of the reported associations.

Several limitations should be acknowledged. First, content validity was evaluated by a small expert panel (*n* = 2), which may limit the stability of the CVI estimates despite meeting established agreement thresholds. Second, the dichotomization of practice scores, while analytically useful, may have reduced variability and resulted in potential loss of information. Third, the use of non-probability convenience sampling, combined with the higher proportion of female participants, may limit generalizability to the broader Taiwanese adult population. In addition, no sampling weights were applied to adjust for population distribution, which may further constrain the representativeness of the findings. Self-reported behavioral measures may also be subject to recall bias and social desirability bias.

Several knowledge items exhibited ceiling effects, which may have reduced score variability and limited sensitivity in detecting associations. The comparatively lower reliability observed in the knowledge domain suggests that knowledge-related findings should be interpreted with caution and may reflect the multidimensional and formative characteristics of factual constructs. Certain items were excluded from inferential modeling due to unsatisfactory reliability and/or ceiling effects, which may have slightly narrowed construct coverage within composite scores.

Future research employing probability-based sampling, objective behavioral indicators, confirmatory factor analysis, and longitudinal or intervention-based designs is warranted to further validate and extend these findings.

## 5. Conclusions

This study developed and evaluated a culturally adapted sustainable diet KAP questionnaire for Taiwanese adults, providing evidence of acceptable content validity, internal consistency for the attitude and practice domains, and temporal stability at the item level. The findings indicate that attitudinal factors were more consistently associated with low-carbon dietary adherence than knowledge scores within this cross-sectional sample.

The instrument provides a structured and culturally relevant framework for assessing sustainable diet-related knowledge, attitudes, and practices in population-based research. By distinguishing between psychological, sociodemographic, and behavioral correlates, the questionnaire may facilitate more nuanced analyses of determinants of sustainable dietary behavior.

Future longitudinal and intervention studies are warranted to clarify causal pathways and to further examine the applicability of this instrument in more diverse and representative populations. Together, these findings contribute to the methodological foundation for sustainable diet research in Asian contexts and highlight the relevance of attitudinal dimensions in shaping dietary behavior within this analytical framework.

## Figures and Tables

**Table 1:** Factor loadings, explained variance, and reliability indices of the knowledge, attitude, and practice (KAP) domains for a sustainable diet.

Domain K		Domain A		Domain P	
Guttman’s λ = 0.576		Cronbach’s α = 0.853		Cronbach’s α = 0.834	
Variance = 10.7%		Variance = 16.4%		Variance = 15.9%	
Item	Factor loadings	Item	Factor loadings	Item	Factor loadings
K1	0.613	A1	0.685	P1	0.624
K2	0.296	A2	0.659	P2	0.710
K3	0.139	A3	0.738	P3	0.705
K4	0.594	A4	0.556	P4	0.679
K5	0.488	A5	0.721	P5	0.803
K6	0.607	A6	0.788	P6	0.645
K7	0.671	A7	0.769	P7	0.631
K8	0.618				
K9	0.342				

Notes: 1. Exploratory factor analysis was conducted separately for the knowledge (K), attitude (A), and practice (P) domains using principal axis factoring with varimax rotation. 2. Factor loadings ≥ 0.40 are commonly considered acceptable in exploratory factor analysis; lower loadings were retained when theoretically justified and supported by prior content validity assessment. 3. Explained variance represents the percentage of variance explained by the extracted factor within each domain. 4. Internal consistency was assessed using Cronbach’s α for the attitude and practice domains, and Guttman’s λ for knowledge domains. 5. Each item was allowed to load on only one factor.

**Table 2:** Item-level test–retest reliability of the knowledge, attitude, and practice (KAP) domains of the sustainable diet questionnaire.

Knowledge Domain ^1,3,4^			Attitude Domain ^2,4^			Practice Domain ^4^		
Item	Cohen’s Kappa (κ)	*p*	Item	ICC (95%-CI)	*p*	Item	ICC (95%-CI)	*p*
K1	0.245	0.050	A1	0.571 (0.288–0.741)	0.001 *	P1	0.793 (0.659–0.875)	<0.001 *
K2	0.270	0.030 *	A2	0.337 (−0.072–0.593)	0.047 *	P2	0.623 (0.378–0.772)	<0.001 *
K3	0.316	0.012 *	A3	0.468 (0.116–0.679)	0.008 *	P3	0.497 (0.174–0.694)	0.004 *
K4	0.659	<0.001 *	A4	0.623 (0.381–0.771)	<0.001 *	P4	0.789 (0.652–0.872)	<0.001 *
K5	0.633	<0.001 *	A5	0.546 (0.257–0.724)	0.001 *	P5	0.534 (0.241–0.716)	0.001 *
K6 ^5^	-	-	A6	0.587 (0.317–0.751)	<0.001 *	P6	0.601 (0.339–0.759)	<0.001 *
K7	0.416	<0.001 *	A7	0.372 (−0.038–0.620)	0.035 *	P7	0.595 (0.334–0.754)	<0.001 *
K8	0.174	0.159 *						
K9	0.204	0.089 *						

Notes: ^1^ Items K3 and K9 are multiple-response questions. Items demonstrating κ < 0.30, non-estimable κ, or non-significant agreement were excluded from composite score calculation but retained for descriptive reporting. ^2^ Items A2 and A7 exhibited ICC 95% confidence intervals crossing zero and were excluded from composite score calculation. ^3^ Knowledge items were assessed using Cohen’s kappa (κ); attitude and practice items were assessed using intraclass correlation coefficients (ICC; two-way mixed-effects model, absolute agreement). ^4^ Interpretation of reliability coefficients followed established methodological guidelines [17,18]. ^5^ Item K6 exhibited no response variability (all participants selected the same response); therefore, Cohen’s kappa was not estimable. * Indicates a statistically significant difference (*p* < 0.05).

**Table 3:** Sociodemographic and lifestyle characteristics of the participants (N = 587).

Category	Variable	*n* (%)
Gender ^1^	Male	218 (37.1)
	Female	359 (61.2)
Age	18–23	153 (26.1)
	24–35	149 (25.4)
	36–51	151 (25.7)
	52–65	134 (22.8)
Education level	High school/vocational school and below	40 (6.8)
	College/university	363 (61.8)
	Graduate school or above	184 (31.3)
Occupation	Student	170 (29.0)
	Full-time job	318 (54.2)
	Others ^2^	99 (16.9)
Residential area	Northern	465 (79.2)
	Central	58 (9.9)
	Southern	44 (7.5)
	Eastern and Offshore islands	20 (3.4)
Monthly income (NT$) ^3^	Below 20,000	128 (23.9)
	20,000–<40,000	120 (22.4)
	40,000–<60,000	153 (28.6)
	Above 60,000	134 (25.0)
Frequency of eating out	≤1 time/day	178 (30.3)
	2 times/day	196 (33.4)
	≥3 times/day	213 (36.3)
Eating out category ^4^	Breakfast	285 (48.6)
	Lunch	454 (77.3)
	Dinner	404 (68.8)
	Snacks/supper	152 (25.9)
Awareness of sustainability in Taiwan’s Dietary Guidelines	Yes, aware	134 (22.8)
	Yes, unsure	204 (34.8)
	No	239 (40.7)

Notes: ^1^ Percentages may not sum to 100% due to missing responses (gender missing, *n* = 10). ^2^ “Others” include part-time workers, freelancers, homemakers, and retirees. ^3^ Income percentages were calculated based on valid responses (*n* = 535; 52 missing). ^4^ The eating-out category was assessed as a multiple-response item; therefore, percentages may exceed 100%.

**Table 4:** Comparisons of knowledge, attitude, and practice (KAP) scores according to sociodemographic and lifestyle characteristics of the participants (N = 587) ^1,2^.

Characteristic	Knowledge	Attitude	Practice
Total	6.4 ± 1.46	28.8 ± 4.60	23.4 ± 5.22
Gender			
Male (*n* = 218)	6.1 ± 1.65 *	27.9 ± 4.98 *	23.1 ± 5.18
Female (*n* = 359)	6.5 ± 1.29	29.3 ± 4.12	23.6 ± 5.19
Age (years)			
18–23 (*n* = 153)	6.3 ± 1.58	27.5 ± 4.63 ^c^	21.4 ± 5.35 ^c^
24–35 (*n* = 149)	6.4 ± 1.36	28.5 ± 4.44 ^bc^	23.7 ± 5.25 ^b^
36–51 (*n* = 151)	6.5 ± 1.23	29.0 ± 4.57 ^b^	23.0 ± 4.83 ^b^
52–65 (*n* = 134)	6.3 ± 1.66	30.3 ± 4.34 ^a^	25.8 ± 4.43 ^a^
Education Level			
High school/vocational school and below (*n* = 40)	4.9 ± 2.00 ^b^	27.9 ± 5.81 ^b^	22.5 ± 5.18 ^b^
College/university (*n* = 363)	6.5 ± 1.36 ^a^	28.4 ± 4.47 ^ab^	22.9 ± 5.21 ^b^
Graduate school or above (*n* = 184)	6.5 ± 1.35 ^a^	29.7 ± 4.45 ^a^	24.6 ± 5.07 ^a^
Occupation			
Student (*n* = 170)	6.5 ± 1.51 ^a^	27.8 ± 4.56 ^b^	21.0 ± 5.25 ^c^
Full-time job (*n* = 318)	6.4 ± 1.43 ^ab^	28.9 ± 4.58 ^a^	24.0 ± 4.86 ^b^
Others ^2^ (*n* = 99)	6.1 ± 1.44 ^b^	29.9 ± 4.43 ^a^	25.6 ± 4.81 ^a^
Residential area			
Northern (*n* = 465)	6.4 ± 1.45 ^b^	29.0 ± 4.60	23.1 ± 5.12
Central (*n* = 58)	6.3 ± 1.46 ^b^	28.1 ± 4.67	24.1 ± 5.84
Southern (*n* = 44)	5.9 ± 1.37 ^b^	28.4 ± 3.73	24.7 ± 5.00
Eastern and offshore islands (*n* = 20)	7.1 ± 1.61 ^a^	27.1 ± 5.78	25.0 ± 5.61
Monthly income			
Below 20,000 (*n* = 128)	6.6 ± 1.39 ^a^	28.0 ± 4.53 ^c^	21.4 ± 5.38 ^c^
20,000–<40,000 (*n* = 120)	6.3 ± 1.48 ^ab^	28.3 ± 4.53 ^bc^	23.3 ± 4.73 ^b^
40,000–<60,000 (*n* = 153)	6.4 ± 1.33 ^ab^	29.2 ± 4.22 ^ab^	24.3 ± 5.26 ^ab^
Above 60,000 (*n* = 134)	6.2 ± 1.64 ^b^	29.6 ± 4.97 ^a^	24.7 ± 4.88 ^a^
Frequency of eating out			
≤1 time/day (*n* = 178)	6.5 ± 1.25	29.5 ± 4.34 ^b^	25.2 ± 4.55 ^a^
2 times/day (*n* = 196)	6.4 ± 1.59	28.7 ± 4.44 ^ab^	23.0 ± 4.94 ^b^
≥3 times/day (*n* = 213)	6.3 ± 1.51	28.2 ± 4.89 ^a^	22.3 ± 5.63 ^b^
Eating out category			
Breakfast (*n* = 285)	6.5 ± 1.43	28.6 ± 4.56	23.5 ± 5.42
Lunch (*n* = 454)	6.5 ± 1.41	28.7 ± 4.72	23.5 ± 5.17
Dinner (*n* = 404)	6.5 ± 1.42	28.8 ± 4.51	23.4 ± 5.25
Snacks/Supper (*n* = 152)	6.3 ± 1.57	28.1 ± 4.72	24.8 ± 5.11

Notes: ^1^ Data are presented as mean ± standard deviation (SD). ^2^ For variables with more than two groups, one-way analysis of variance (ANOVA) was performed, followed by Bonferroni-adjusted post hoc comparisons. Different superscript letters indicate statistically significant differences between groups (*p* < 0.05). * Indicates a statistically significant difference between two groups based on an independent-samples t-test (*p* < 0.05).

**Table 5:** Multivariable logistic regression analysis of factors associated with low adherence to a low-carbon diet (N = 587).

Variable	Adjusted OR (95% CI)	*p*-Value
**Psychological Factors**		
Knowledge score	0.997 (0.840–1.182)	0.969
Attitude score	0.915 (0.874–0.957)	<0.001 *
**Sociodemographic and Behavioral Factors**		
Education Level		
Graduate School or above	1.00 (Ref.)	
College/University or below	0.967 (0.505–1.853)	0.919
Monthly Income (TWD)		
≥40,000	1.00 (Ref.)	
<40,000	0.749 (0.360–1.557)	0.439
Eating Out Frequency		
>3 times/day	1.00 (Ref.)	
≤2 times/day	0.476 (0.279–0.814)	0.007 *
**Policy Awareness**		
Sustainability Awareness in Guidelines		
Lack awareness	1.00 (Ref.)	
Clear awareness	0.373 (0.146–0.950)	0.039 *

Notes: Dependent variable: Low adherence to a low-carbon diet (≤2). OR = Odds Ratio; CI = Confidence Interval; Ref. = Reference group. An OR < 1 indicates a protective effect (i.e., factors associated with a reduced likelihood of having low adherence). All variables were entered into the fully adjusted model simultaneously, adjusted for gender, age, residential area, occupation, and all variables listed above. * Indicates a statistically significant association (*p* < 0.05).

## Data Availability

The data presented in this study are available on request from the corresponding author due to ethical and privacy restrictions related to human participant data.

## Supplementary Materials

The following supporting information can be downloaded at: https://www.mdpi.com/article/10.3390/nu18060908/s1, Table S1: Item-level responses to knowledge questions on sustainable diets among participants (N = 587); Table S2: Item-level responses to attitude statements on sustainable diets among participants (N = 587); Table S3: Item-level responses to practice items related to sustainable diets among participants (N = 587); Table S4: Correlations between knowledge, attitudes, and practices across sociodemographic and behavioral characteristics; Table S5: Model Diagnostics for Multivariable Logistic Regression Analyses Examining Factors Associated with Low Adherence to a Low-Carbon Diet (N = 587); Table S6: Inter-Item Correlation Matrix for the Knowledge Domain.

## Abbreviations

ANOVA	Analysis of Variance
CVI	Content Validity Index
EFA	Exploratory Factor Analysis
FAO	Food and Agriculture Organization
ICC	Intraclass Correlation Coefficient
I-CVI	The item-level content validity index
KAP	Knowledge, Attitudes and Practices
S-CVI/Ave	Scale-level content validity index based on the average method
S-CVI/UA	Scale-level content validity index based on the universal agreement method
