# Supplementary material for: Development and Psychometric Validation of a Knowledge, Attitudes, and Practices (KAP) Questionnaire on Sustainable Diets in Taiwan
Source: Nutrients. 2026 Mar 13;18(6):908. doi: 10.3390/nu18060908 (PMC13028838; doi:10.3390/nu18060908)
Supplement: Supplementary file 1 [file nutrients-18-00908-s001.zip › nutrients-4161990-supplementary.pdf]

Table S1. Item-level responses to knowledge questions on sustainable diets among participants (N = 587).

Category	Variable	n (%)[1]
Which of the following is NOT a characteristic of environmentally friendly diets (sustainable diets)?	Reducing meat consumption	16 (2.7)
	Choosing local ingredients	15 (2.6)
	Increasing consumption of processed food†	545 (92.8)
	Reducing food waste	11 (1.9)
Which type of diet has the least environmental impact?	High meat, low fruits, and vegetables	39 (6.6)
	Balanced diet	277 (47.2)
	Vegan diet†	204 (34.8)
	Not sure	67 (11.4)
What are the main focuses of "choosing environmentally friendly diets (sustainable diets)"? (Please select three)[2]	Environmental impact†	542 (92.3)
	Nutritional balance†	434 (73.9)
	Cultural adaptability	169 (28.8)
	Economic feasibility	386 (65.8)
	Social equity†	139 (23.7)
Which foods typically produce the highest greenhouse gas emissions during production?	Vegetables	8 (1.4)
	Fruits	3 (0.5)
	Beef†	556 (94.7)
	Legumes	20 (3.4)
What does the concept of "Farm to Table" primarily emphasize?	The freshness of food	105 (17.9)
	The environmental impact of the entire process of food production and consumption†	469 (79.9)
	The efficiency of farm production	10 (1.7)
	The cooking techniques of restaurants	3 (0.5)
What does "Eating fish while caring for ocean health (sustainable fisheries)" mainly focus on?	The freshness of fish	15 (2.6)
	The price of fish	10 (1.7)
	Maintaining the balance of fish populations and the health of marine ecosystems†	561 (95.6)
	Increasing fish catch volume	1 (0.2)
What does the concept of "Food Miles" primarily emphasize?	The shelf life of food	26 (4.4)
	The transportation distance of food from its origin to the consumers†	513 (87.4)
	The price of food	8 (1.4)
	The nutritional value	40 (6.8)
Which of the following is NOT a health benefit of choosing environmentally friendly diets (sustainable diets)?	Increased fruit and vegetable intake	10 (1.7)
	Reduced consumption of processed foods	28 (4.8)
	Increased meat consumption†	538 (91.7)
	Increased dietary fiber intake	11 (1.9)
Please select the Taiwan agricultural products that represent "growing healthy food while protecting land and water resources (sustainable agriculture)" (multiple choices are allowed)[2]	Organic rice†	486 (82.8)
	Vegetables sold directly by small farmers†	473 (80.6)
	Locally farmed fish†	377 (64.2)
	Not sure	15 (2.6)
	Other	1 (0.2)

Notes:

[1] Data are presented as a number (percentage).

[2] Items marked as multiple-response allowed more than one option to be selected; therefore, percentages may exceed 100%.

† Indicates the correct answer.

Table S2. Item-level responses to attitude statements on sustainable diets among participants (N = 587).

Category	Variable	n (%)
I am willing to modify my eating habits for the sake of environmental sustainability.	Strongly disagree	6 (1.0)
	Disagree	27 (4.6)
	Neutral	133 (22.7)
	Agree	284 (48.4)
	Strongly agree	137 (23.3)
Choosing local ingredients supports local agriculture and reduces carbon emissions.	Strongly disagree	9 (1.5)
	Disagree	26 (4.4)
	Neutral	69 (11.8)
	Agree	219 (37.3)
	Strongly agree	264 (45.0)
Schools should incorporate environmentally friendly diets (sustainable diets) into the nutrition education curriculum.	Strongly disagree	8 (1.4)
	Disagree	9 (1.5)
	Neutral	74 (12.6)
	Agree	206 (35.1)
	Strongly agree	290 (49.4)
Reducing meat consumption benefits both the environment and health.	Strongly disagree	8 (1.4)
	Disagree	53 (9.0)
	Neutral	139 (23.7)
	Agree	212 (36.1)
	Strongly agree	175 (29.8)
Food labels should include information about carbon emissions (carbon footprint).	Strongly disagree	8 (1.4)
	Disagree	20 (3.4)
	Neutral	112 (19.1)
	Agree	201 (34.2)
	Strongly agree	246 (41.9)
Everyone has a responsibility to protect the environment through dietary choices.	Strongly disagree	11 (1.9)
	Disagree	16 (2.7)
	Neutral	76 (12.9)
	Agree	227 (38.7)
	Strongly agree	257 (43.8)
Technological innovations (e.g., food traceability systems and sustainability certification labels) can help me practice sustainable eating.	Strongly disagree	5 (0.9)
	Disagree	20 (3.4)
	Neutral	77 (13.1)
	Agree	218 (37.1)
	Strongly agree	267 (45.5)

Notes:

Data are presented as a number (percentage). Responses were measured on a five-point Likert scale ranging from strongly disagree to agree strongly.

Table S3. Item-level responses to practice items related to sustainable diets among participants (N = 587).

Category	Variable	n (%)
I consume at least three servings of vegetables daily (1 serving equals 100 grams raw or 1/2 - 2/3 bowl cooked).	Never	14 (2.4)
	Rarely	97 (16.5)
	Sometimes	196 (33.4)
	Often	199 (33.9)
	Always	81 (13.8)
I avoid consuming processed foods or sweets.	Never	35 (6.0)
	Rarely	125 (21.3)
	Sometimes	178 (30.3)
	Often	172 (29.3)
	Always	77 (13.1)
I choose protein sources with lower environmental impact (e.g., legumes).	Never	32 (5.5)
	Rarely	95 (16.2)
	Sometimes	189 (32.2)
	Often	190 (32.4)
	Always	81 (13.8)
I control my salt intake in food.	Never	17 (2.9)
	Rarely	75 (12.8)
	Sometimes	147 (25.0)
	Often	224 (38.2)
	Always	124 (21.1)
I select food items that align with low-carbon diets.	Never	30 (5.1)
	Rarely	96 (16.4)
	Sometimes	229 (39.0)
	Often	174 (29.6)
	Always	58 (9.9)
I purchase local organic products or items directly sold by small farmers in Taiwan.	Never	23 (3.9)
	Rarely	72 (12.3)
	Sometimes	200 (34.1)
	Often	215 (36.6)
	Always	77 (13.1)
I use technological tools (e.g., agricultural traceability platforms, Taiwan Green Mark, and ingredient mapping apps) to track food sources and sustainability certifications.	Never	57 (9.7)
	Rarely	98 (16.7)
	Sometimes	186 (31.7)
	Often	175 (29.8)
	Always	71 (12.1)

Notes:

Data are presented as a number (percentage). Responses were measured on a five-point Likert scale ranging from "never" to "always".

Table S4. Correlations between knowledge, attitudes, and practices across sociodemographic and behavioral characteristics[1].

Variable	Knowledge-Attitude		Knowledge-Practice		Attitude-Practice	
	r	*p*	*r*	*p*	*r*	*p*
**Gender[2]**						
Male (n=218)	0.273	<0.001*	0.106	0.119	0.513	<0.001*
Female (n=359)	0.008	0.876	-0.050	0.342	0.379	<0.001*
**Age**						
≤35 (n=302)	0.265	<0.001*	0.028	0.628	0.438	<0.001*
>35 (n=285)	0.085	0.154	0.059	0.322	0.432	<0.001*
**Education level**						
High school or vocational school and below (n=40)	0.269	0.094	0.218	0.177	0.565	<0.001*
College/University (n=363)	0.174	0.001*	-0.057	0.277	0.393	<0.001*
Graduate school or above (n=184)	0.111	0.133	0.135	0.067	0.504	<0.001*
**Occupation**						
Student (n=170)	0.252	0.001*	-0.001	0.990	0.456	<0.001*
Full-time job (n=318)	0.205	<0.001*	0.149	0.008*	0.393	<0.001*
Others[2] (n=99)	0.044	0.669	-0.064	0.530	0.506	<0.001*
**Residential area**						
Northern (n=465)	0.171	<0.001*	-0.005	0.912	0.439	<0.001*
Central (n=58)	0.227	0.086	0.132	0.323	0.516	<0.001*
Southern (n=44)	-0.160	0.301	0.050	0.749	0.553	<0.001*
Eastern and Offshore islands (n=20)	0.775	<0.001*	0.832	<0.001*	0.696	0.001*
**Monthly income (NT$)[2]**						
Below 20,000 (n=128)	0.174	0.049*	-0.154	0.083	0.424	<0.001*
20,000- <40,000 (n=120)	0.188	0.039	0.155	0.090	0.306	0.001*
40,000- <60,000 (n=153)	0.166	0.040*	0.127	0.119	0.443	<0.001*
Above 60,000 (n=134)	0.188	0.030*	0.125	0.151	0.576	<0.001*
**Eating out frequency**						
≤1 time/day (n=178)	0.116	0.122	0.085	0.258	0.506	<0.001*
2 times/day (n=196)	0.172	0.016*	-0.047	0.515	0.364	<0.001*
≥3 times/day (n=213)	0.206	0.002*	0.062	0.369	0.459	<0.001*

**Eating out category[3]**						
Breakfast (n=285)	0.087	0.144	0.057	0.339	0.366	<0.001*
Lunch (n=454)	0.167	<0.001*	0.022	0.642	0.438	<0.001*
Dinner (n=404)	0.166	0.001*	-0.008	0.877	0.434	<0.001*
Supper (n=152)	0.307	<0.001*	0.133	0.102	0.422	<0.001*
**Awareness of Sustainability in Taiwan's Dietary Guidelines and Indicators**						
Yes, aware	0.164	0.059	0.213	0.014	0.511	<0.001*
Yes, unsure	0.151	0.030*	0.043	0.541	0.414	<0.001*
No	0.210	0.001*	-0.007	0.909	0.392	<0.001*

Notes:

[1] Data were analyzed using Pearson's correlation coefficient (r).

[2] Data include participants who declined to report gender (n = 10) or monthly income (n = 52).

[3] Multiple responses were allowed for eating-out categories.

* indicates a statistically significant association ($p < 0.05$).

Table S5 Model Diagnostics for Multivariable Logistic Regression Analyses Examining Factors Associated with Low Adherence to a Low-Carbon Diet (N = 587)

Model	Variables Included	Nagelkerke R^2	Hosmer–Lemeshow χ^2 (p-value)	AUC (95% CI)	VIF Range
**Model 1**	Education, monthly income, sustainability awareness, and eating-out frequency	0.159	8.593 (p = 0.283)	0.716 (0.665-0.767)	1.023-2.213
**Model 2**	Model 1 + age and gender	0.208	13.417 (p = 0.098)	0.750 (0.704-0.795)	1.023-2.213
**Model 3**	Model 2 + residential area, occupation, knowledge score, and attitude score	0.246	10.774 (p = 0.215)	0.778 (0.734-0.821)	1.023-2.213

Notes

1. Dependent variable: Low adherence to a low-carbon diet (≤ 2).
2. Model 1 included sociodemographic predictors (education, monthly income), sustainability awareness, and eating-out frequency.
3. Model 2 additionally adjusted for age and gender.
4. Model 3 further adjusted for residential area, occupation, knowledge score, and attitude score.
5. Multicollinearity was assessed using variance inflation factors (VIF); values < 5 were considered indicative of no problematic collinearity.
6. Model calibration was evaluated using the Hosmer–Lemeshow goodness-of-fit test; a non-significant p-value ($p > 0.05$) indicates adequate model fit.
7. Discriminatory capacity was assessed using receiver operating characteristic (ROC) curve analysis and the area under the curve (AUC).

Table S6 Inter-Item Correlation Matrix for the Knowledge Domain

Item	K1	K2	K3	K4	K5	K6	K7	K8	K9
K1	1								
K2	0.119	1							
K3	0.058	0.009	1						
K4	0.230	0.172	0.042	1					
K5	0.125	0.080	0.070	0.186	1				
K6	0.229	0.088	0.064	0.208	0.285	1			
K7	0.273	0.126	0.055	0.323	0.232	0.367	1		
K8	0.418	0.091	0.066	0.259	0.125	0.234	0.275	1	
K9	0.157	0.069	0.031	0.109	0.132	0.142	0.057	0.192	1

Notes

1. Pearson correlation coefficients are reported.
2. Values < 0.15 suggest a weak inter-item association.
3. Values between 0.15-0.50 indicate acceptable item relationships.
4. No correlations exceeded 0.50, suggesting no evidence of item redundancy.
